# Participants' experiences of the management of screen‐detected complex polyps within a structured bowel cancer screening programme

**DOI:** 10.1111/hex.13525

**Published:** 2022-07-07

**Authors:** Lenira Semedo, Ardiana Gjini, Sunil Dolwani, Kate J. Lifford

**Affiliations:** ^1^ Division of Population Medicine School of Medicine, Cardiff University Cardiff UK; ^2^ Public Health Wales Wales UK

**Keywords:** colon polyp, complex polyps, patient experience, quality of life

## Abstract

**Background:**

The Bowel Screening Wales complex polyp removal service was introduced to address variations in surgery rates for screen‐detected complex benign colorectal polyps, to improve the quality of the screening service and to make management of these polyps more equitable across Wales. Little is known about patient experiences and the potential impact on quality of life when undergoing complex polyp removal. This study is part of a wider research programme evaluating the decision‐making, pathways and outcomes from complex polyp removal.

**Objective:**

This study aimed to understand experiences of having a complex polyp removed and how this may influence quality of life.

**Design:**

Semi‐structured telephone interviews were conducted, and a thematic approach was used for data analysis.

**Setting and Participants:**

All participants had a complex polyp removed after a positive stool test and review by Bowel Screening Wales' Network Multi‐Disciplinary Team.

**Results:**

Twenty‐one participants were interviewed. Most participants had their complex polyps removed endoscopically and reported no or minor problems or negative outcomes following their procedure. For a small minority, worse problems (e.g., pain, bowel dysfunction) and negative outcomes (e.g., cancer) followed their procedures. Most participants felt supported and reassured throughout their procedures. Any physical and emotional changes to quality of life were mainly linked to procedure outcomes.

**Discussion:**

Experiences of complex polyp removal were generally positive, with minimal changes in quality of life.

**Conclusions:**

While most people had a positive experience of having a complex polyp removed, support initiatives, such as counselling or signposting to coping strategies, may be helpful to reduce any potential negative effects of procedures on quality of life.

**Patient or Public Contribution:**

Four patient and public involvement partners provided feedback on participant materials.

## 1 BACKGROUND

Colorectal cancer (CRC) is the fourth most common cancer in the United Kingdom, with over 42,000 new cases diagnosed per year.^1^ Bowel cancer screening aims to reduce CRC mortality through early diagnosis and removal of precancerous polyps found in the colon. In the United Kingdom, screening is implemented for those aged 50–74 years (varying by individual country) using biennial stool tests. Following a positive stool test, participants are invited for colonoscopy to determine the cause of the positive test. At colonoscopy, approximately 2.1% of the polyps identified are ‘complex polyps’^2^ or ‘significant polyps’.^3^ They are complex because of characteristics (e.g., size, morphology, location, access) that make them difficult and more risky to remove.^4^, ^5^ Indeed, until developments of advanced endoscopic techniques, surgical removal was the only option.^4^ Endoscopic removal of complex polyps is associated with less risk and is cost‐effective, but is dependent on endoscopic skill.^6^, ^7^, ^8^, ^9^, ^10^

There is a dearth of evidence on patient experience and quality of life in relation to complex removal of polyps. A comparison of endoscopic polyp removal and sigmoid resection for colon cancer showed more bowel dysfunction symptoms following sigmoid resection.^11^ While there was no difference in overall quality of life between the two procedure groups, those who underwent a sigmoid resection had greater impairment in quality of life due to bowel dysfunction.^11^ Bowel dysfunction due to surgery has been previously suggested to have a long‐term negative impact on patients' quality of life.^12^ Miller and Sedgewick^13^ compared patient experiences of polyp cancers within the English Bowel Cancer Screening Programme by procedure type, endoscopy or surgery. A difference in quality‐of‐life issues around self‐care was found (but the direction of effect was not reported). Interviews with a small subsample found that both groups of patients experienced fear and anxiety, however, those who underwent surgery received more clinical support than those who underwent an endoscopy procedure.^13^ This is expected because NHS support processes for patients after endoscopic procedures differ from the support provided to surgically managed patients; surgical postoperative patients have regular follow‐up and support when compared to endoscopic patients, who only have one point of contact.

The Bowel Screening Wales complex polyp removal service was established in 2011 as a pilot and integrated into the service in 2017. It was introduced to address variation in rates of surgery^2^ and high incomplete resection rates as well as to make the screening service more equitable across Wales. The complex polyp removal service is comprised of a Network Multidisciplinary Team (NMDT), National Referral Centre (NRC) and central co‐ordination, and has defined specific referral criteria. The patient's local team is encouraged to attend the virtual NMDT meeting, where case images and videos are reviewed and discussed and a decision on management is made. Where appropriate, cases can be referred on to the NRC for expert treatment. In contrast to local centres, the NRC offers a number of different advanced endoscopic procedures. A small (*n* = 24) evaluation of the NMDT and NRC pilot in Wales showed that participants wanted to have the decision to be referred to the NMDT discussed with them and most had treatment options discussed with them after the NMDT. The majority of participants described their experience of the NRC as good or excellent.^14^

The premise of the complex polyp removal service is that having an endoscopic procedure will be more cost‐effective and will lead to better outcomes. Examination of the quantitative outcomes and health economics of bowel screening participants with complex polyps detected at screening colonoscopy has been completed (to be reported in a separate manuscript). While those outcomes are clinical (e.g., complications, hospital stays), it is also important to assess patient experience and quality of life. As clinical and patient experience outcomes are likely linked, we expected that those who had an endoscopic procedure may have a better experience of the complex polyp removal service than those who had a surgical procedure. However, much of the literature around quality of life and different procedures suggests limited difference. The aim of this study was, therefore, to explore patient experience of complex polyp removal and how it might influence quality of life.

## 2 METHODS

### 2.1 Sample

All patients who went through the complex polyp removal service and had a complex polyp removed within a 12‐month period (March 2019–2020) were sent a study invitation pack (cover letter, participant information sheet, questionnaire and consent forms) in September 2020. A reminder letter was sent approximately 4 weeks after the initial invitation if no reply had been received. At the time participants would have had a complex polyp removed (March 2019–2020), bowel screening was available to people in Wales aged between 60 and 74 years.

### 2.2 Procedure

Participants were able to choose whether to take part in an interview and/or complete a questionnaire for the study. The questionnaire data were collected primarily for a health economics analysis (to be reported elsewhere), but are used within the present study to describe the sample. The questionnaire consisted of the EQ‐5D‐5L^15^, ^16^; https://euroqol.org/eq-5d-instruments/eq-5d-5l-about/ along with questions about demographic background (age, education, employment and living arrangements). The EQ‐5D‐5L is based on two components: the utility index and a vertical visual analogue scale (VAS); the latter is reported here. The VAS asks ‘how good or bad your health is today’, and has a range of 0 (worst health you can imagine) to 100 (best health you can imagine).

Upon receiving a completed consent form for a telephone interview, a researcher from the study team contacted the participant to arrange an interview and answer any questions. At the interview appointment, following consent, participants were asked to describe their experience of going for a complex polyp procedure narratively (from being referred following positive result, to outcomes in following weeks). Interviews were semi‐structured, and topics included procedure details, experience of procedure (including practical issues like travel arrangement, friends/family support), difficulties arising from the procedure, current quality of life and perceived influence of procedure (including outcomes) on quality of life (see the Supporting Information File for topic guide). Prompts around quality of life included domains from the EQ‐5D‐5L questionnaire (mobility, self‐care, usual activities, pain/discomfort and anxiety/depression; https://euroqol.org/eq-5d-instruments/eq-5d-5l-about/.^15^, ^16^ Interviews were audio‐recorded and transcribed verbatim. The interviewers were experienced and had a wider team who provided debriefing support where appropriate.

Data about participants' relevant healthcare (e.g., type and location of the procedure) were collected (with participant consent) from Bowel Screening Wales.

### 2.3 Analysis

Transcripts were analysed thematically,^17^ and NVivo 12^18^ was used to manage the data. Analysis of the interview transcripts followed an iterative process to capture participants' reality and draw meaning from their experiences in the context in which they occurred.^17^ After familiarization with the data, a coding framework was generated through discussion, during which 20% of the transcripts were double‐coded. Codes most pertinent to the research questions were then analysed to refine the codes, which resulted in identifying themes. Other codes were checked for relevant information where appropriate. Similarities and differences were explored within the data, both by looking through the codes and using visual maps.

## 3 RESULTS

### 3.1 Sample characteristics

Of the 71 people invited to participate, 21 completed an interview between October and December 2020 (response rate 30%). One participant chose not to take part in the questionnaire study, so we do not have demographic data for them. Participants were aged between 61 and 74 years (mean = 67.7, SD = 3.9), and the majority of participants were male (76%; Table 1). Most of the participants were retired (71%), and the majority owned their own home (81%; Table 1). The majority of participants (86%) had undergone an endoscopic procedure to remove their polyp. Two participants had surgery to remove their polyp, and one participant went through an endoscopic procedure, followed by surgery for complete removal of their polyp (Table 1). Most participants rated their current quality of life as quite good, with a mean VAS score of 78.9 (SD = 16.0).

**Table 1:** Participant characteristics.

	*N* (%) or mean (SD)
Age in years	67.7 (3.9)
Missing	1
Sex	
Female	6 (29%)
Male	15 (71%)
Highest level of education	
Finished school at or before age of 15	5 (24%)
No qualifications/left school at 16	3 (14%)
Completed certificate of secondary educations, O‐levels or equivalent	2 (10%)
Completed A levels or equivalent	4 (19%)
Completed further education but not degree	2 (10%)
Completed a Bachelor's degree/Master's/PhD	3 (14%)
Other (full certificate)	1 (5%)
Missing	1 (5%)
Employment	
Employed full‐time	4 (19%)
Employed part‐time	1 (5%)
Retired	15 (71%)
Missing	1 (5%)
Home ownership	
Own outright	17 (81%)
Own mortgage	2 (10%)
Rent from local authority/housing association	1 (5%)
Missing	1 (5%)
Procedure type/location	
Endoscopy	18 (86%)
Local Assessment Centre	7
National Referral Centre	11
Surgery	2 (10%)
Local Assessment Centre	2
Endoscopy followed by surgery	1 (5%)
National Referral Centre	1
Findings	
Cancer diagnosis	2 (10%)

### 3.2 Themes

Participants' narratives describe their experiences through different stages of the procedure (before, during and after) and how they adapted to physical and emotional challenges. Participants with uncomplicated procedures and positive outcomes reported a better experience and quality of life. Negative changes in quality of life after polyp removal were reported by a small minority who had complicated procedures and experienced problems and negative outcomes.

Participant quotes are used to portray the themes. Individual quotes are italicized and identified by the participant study number (e.g., P1), type of procedure (End = endoscopy, Surg = surgery or End‐Surg = endoscopy followed by surgery) and location of procedure (NRC = National Referral Centre or LAC = Local Assessment Centre). Three dots within quotes indicate that part of the quote has been removed. Text in brackets (not italicized) is used to add clarity to the quotes and a semicolon within the same quote is used to identify another relevant quote from elsewhere in the transcript reflecting the same theme. Where applicable, participant quotes are represented in Table 2, and a reference is provided in the text using parentheses.

**Table 2:** Participant quotes

Main themes (bold) and subthemes (in italics)	Example quotes	Text reference
**Preparing for the complex polyp removal** *Bowel preparation*	*…one of the worst things about having this procedure was the fact that you had to take this, there's no other way to describe it but god‐awful medicine to clear out your bowel, and that is by far and away the worst thing that I had to experience…* [P46‐End‐NRC] *…you have to keep running to the loo; and then of course all the way, if you have to travel from home to the hospital …you're worried you're going to need the loo again …* [P14‐End‐NRC]	A B
*Travel*	*…so it's about ten miles …so close for us; … public transport's non‐existent here, so it was car anyway*. [P16‐End‐LAC]	C
**Tangible experiences of complex polyp removal** *Pain and discomfort*	*…once the sedative was administered because it was administered while I was in the procedure, the full sedative, things calmed down. It was still uncomfortable, still wincing a bit but it was manageable, yes*. [P44‐End‐LAC]	D
*Additional hospital stay*	*…they kept me in another day for safety, you know in case something happened…they were a bit concerned why I was so sore, so they kept me in for another night… so I spent two nights in hospital*. [P15‐ End‐NRC]	E
**Problems experienced following complex polyp removal** *Pain and bleeding*	*…If I had to get up, I'd have to sort of pull myself up the bed, I couldn't bend or nothing like that. It was far too painful…So as long as I sort of laid still…it was okay you know… I was given pain relief every day*. [P18‐Sur‐LAC]	F
*Uncomfortable sensations and changes in bowel habits*	*… but the only real issue I have now is if I do go to the toilet it seems to take me twice as long to clean myself afterwards*. [P46‐End‐NRC]	G
**Communication and care**	*…their general support, the informed, empathetic support that I've had from the specialist nursing team has been really remarkable…. But when you're at home and you get the call and it is not what you're expecting and you haven't got the chance to put that into context then those broad statements can be, without counselling of any sort …the whole business of breaking news and breaking bad news which is something which is well known and doing that over the phone is always going to be difficult and slightly unsatisfactory; …it's been a rocky road in a way but on the whole the support's been good, to digest that breaking news… is the one thing I feel could be improved*. [P39‐End‐Surg‐NRC]	H
**Reassurance** *Monitoring and further investigations*	*…the polyp has been removed, but at least I'm in the system now…Or you know, somebody's keeping an eye on it*. [P2‐End‐LAC]	I
**The impact of complex polyp removal on QoL**	*…well certainly my mood [changed] after the procedures were finished.… I was …quite, I would say elated. Firstly, when the tumour had gone I think my mood improved anyway and certainly now I would say it's perfectly back to normal*. [P31‐End‐NRC] *… well there are certain things I can't do…at the moment, with this like bending over. stuff like that, you know…cos when I get back up, it does sort of hits me, and I gotta stand still and you know, just hold onto something, in case I fall see…that's the only thing, that's just sort of dragging on at the moment you know…I'm not quite sure if it's a direct result of the surgery …*[P18‐Sur‐LAC] *… I am eating more and better…I wasn't eating very well to be honest with you; I'd lost a lot of weight…when she [wife] saw me because she hadn't been down to see me. I looked like an old man and I could hardly walk…I wasn't allowed to do anything, lift anything; …as I got stronger things got better…sometimes I was really down, the [stoma] bag got me down… and I was unable to do things, unable to go and see my friends; …I am getting stronger every day…the nurses were here every day, it was a bit awkward…it was an open wound. So they came to pack it and re‐dress it every day…It wasn't great*. [P42‐End‐NRC]	J
		K
		L
**Competing factors influencing overall QoL**	*…my quality of life hasn't really altered that much but then my quality of life is more affected by having a 29‐year‐old daughter with special needs than the procedure I had*. [P46‐End‐NRC]	M

Abbreviations: LAC, Local Assessment Centre; NRC, National Referral Centre; QoL, quality of life.

#### 3.2.1 Preparing for the complex polyp removal

##### 3.2.1.1 Worry about undergoing the procedure and its outcomes

All participants had some kind of concern either related to the procedure or its outcome. Part of individuals' experiences of going for a complex polyp removal related to their feelings of anxiety about the procedure being painful and/or worries about a negative outcome (e.g., cancer diagnosis or a stoma bag).

…obviously when I had the test results [positive stool test] … I was rather worried, I was concerned; I thought maybe it was cancer or something big; I suppose I was worried about it being painful … you know having tubes put up your backside and …a camera, … it's a just worrying thought. [P17‐End‐NRC]

##### 3.2.1.2 Bowel preparation

Most participants undergoing an endoscopic procedure drank a solution to cleanse their bowel and described the challenges associated with this. Drinking the solution was difficult, unpleasant and described as one of the worst aspects of having the procedure. A couple of participants said that they would have preferred an alternative (A). For a few individuals, it was not only the drinking of the solution but how it affected them; they reported feeling sore, sick, unwell and ready to faint.

…it causes violent diarrhoea and at one point I was on my own in the house, I thought I was going to pass out… [P39‐End‐Surg‐NRC]

For some participants, taking the bowel preparation caused anxiety about having to go the toilet and travelling to the hospital (B).

Some individuals were already familiar with the bowel preparation process and had no issues doing it. Of the three participants undergoing surgery, only one mentioned having bowel preparation in the form of a suppository. There was no mention of incomplete or poor bowel preparation prior to the procedure.

##### 3.2.1.3 Travel

Whilst some participants had the opportunity to express their preference about which hospital to have the procedure, others did not. Some commented on what may have contributed to decisions about where they had treatment such as specialized support, availability of the hospital setting/healthcare team or timing.

In most cases, the hospital was within easy reach and travel was not an issue (C). Some participants had to travel further to the hospital, which was less practical. Therefore, some booked accommodation or stayed in hospital the night before the procedure.

…they allowed me to go in the night before… I got my son to drop me off… and my daughter came to pick me up… for a personal point of view it was the logistics, although it was a greater distance, the fact I was in the night before, had the procedure and stayed the next night and then came out the following day made life a lot better from my perspective. [P46‐End‐NRC]

#### 3.2.2 Tangible experiences of the complex polyp removal

##### 3.2.2.1 Pain and discomfort

During the procedure, most people talked about experiencing feelings of discomfort, pressure and/or pain with varying degrees of intensity. However, there were a few participants who reported no or very little pain.

…The procedure was no problem at all, slight pain but not too bad at all. [P42‐End‐NRC]

For individuals experiencing higher levels of pain, some commented that pain sensations were attenuated by sedation and/or pain relief, whilst others wanted more pain relief (D). For some of those who experienced more pain, the pain was worse due to other factors (e.g., a fissure and soreness from previous colonoscopy).

##### 3.2.2.2 Watching the polyp being removed

Some participants spoke positively about watching their procedure being done on a screen showing the camera view of the inside of their bowel. This seemed to be a distraction from the procedure, making it more comfortable.

…you can watch it on the screen and it [the polyp] was coming out and he [the surgeon] was putting it in the basket. It was quite interesting actually, yes quite liked that. That took a bit of the pain off. I think it was having something to look at. [P29‐ End‐LAC]

##### 3.2.2.3 Additional hospital stay

Individuals who experienced more difficulties following the procedure (e.g., severe pain or high temperature) stayed in hospital briefly to ensure that they were well before returning home (E).

One extremely serious case required emergency surgery and a lengthier hospital stay of a month.

##### 3.2.2.4 Acceptance

For some participants, there was a sense of accepting that the procedure had to be done and acknowledging some of the complications associated with the procedure.

…It wasn't a good experience, but it was only because physically that's the territory with that sort of surgery… [P39‐End‐Surg‐NRC]

#### 3.2.3 Problems experienced following complex polyp removal

The experience of problems following the procedure varied between individuals in terms of the type of procedure (e.g., endoscopy, surgery or both), significance and time to resolve. A few individuals, including one of those who had undergone surgery, mentioned not experiencing any problems following their procedure.

…To be honest I felt I was fit enough to go back to work the same day because it was done in the morning and I said I could go back to work this afternoon, but they advised me to perhaps take the afternoon off and not go back, just in case, I followed that but there were no complications at all. [P32‐End‐LAC]

##### 3.2.3.1 Pain and bleeding

Some people reported having minor bleeding and pain following the procedure that did not last long.

…I had a little bit of blood… I would say for the first like 2 days… but then I was told, if it persisted, I was to get back in touch with them, but… it stopped after about 2 days… [P3‐End‐NRC]

Others experienced severe pain following the procedure. Of these, for one surgery participant, his pain was restricting and longer lasting and had to be managed with daily medication (F).

##### 3.2.3.2 Uncomfortable sensations and changes in bowel habits

Participants talked about a lingering pressure sensation, making them feel an unpleasant urge to go to the toilet. Of these, a few took medicines to be able to go to the toilet.

…because every time you feel this pressure coming… on your stomach… you think you're going to use the toilet. But nothing happens…it's just continuous wind coming out for a long, long time…. [P28‐End‐NRC]

One individual described how his toilet habits changed after a number of endoscopic procedures were carried out to identify and remove the polyp (G). One surgery participant described having bouts of constipation, followed by diarrhoea, which may have been the effects of cancer treatment.

#### 3.2.4 Negative outcomes of complex polyp removal

##### 3.2.4.1 Cancer

While for most people the polyps removed were benign, biopsies confirmed a cancer diagnosis for a couple of participants, which was then followed by chemotherapy. For one of these, the cancer was contained. However, for the other, there was concern that the cancer may have spread, so further treatment was provided.

…they then did a biopsy of the remaining polyp that showed there was further advanced cancer than they believed… and that there was a small area of tissue in the same area, a small piece of bowel was removed…they were concerned about that the cancer might have spread and I was then referred to oncology for chemotherapy and just as a belt and braces let's not let it spread and do any further damage. [P39‐End‐Sur‐NRC]

##### 3.2.4.2 Other problems identified during the procedure

A couple of participants talked about other problems that were identified during the procedure such as having a stricture or more polyps.

… I'm also appreciative of the fact that they found I had this stricture [narrowing of the intestine]…although it's not related to the polyp…the bleeding, it's all connected;…and I've since been diagnosed with Crohn's. [P2‐End‐LAC]

#### 3.2.5 Communication and care

Most people were very satisfied with the care that they received both during the complex polyp removal and associated procedures. They emphasized the quality of support and advice received as well as effective communication with the healthcare team.

…during the procedures the doctor was there and there was about two or three other nurses and they were there all the time reassuring are you okay and all this sort of stuff. [P32‐End‐LAC]

However, cases of poor care or communication were reported. It is noteworthy that one individual was apprehensive of the procedure because of a comment made by the nurse at the hospital. However, the experience of the procedure and the care received during it meant that this concern was unfounded. One participant, who had undergone surgery for a malignant polyp, praised the support received during his care, but thought that the breaking of bad news could have been handled in a more sensitive way (H).

#### 3.2.6 Support network

Most participants were very grateful to be supported by their families around the time of their procedure.

I have a large family and that's been extremely important to me to feel their support even if it wasn't in person…. and support from my wife as well which has been great. [P39‐End‐Surg‐NRC]

When asked about support from friends/family, a couple of participants reported not receiving specific support, but commented that support was available if they needed it.

#### 3.2.7 Reassurance

Reassurance was a significant aspect linked to the procedure in various ways.

##### 3.2.7.1 Reassurance about polyp being removed and negative test results

Participants reported being reassured that their polyp was removed, that test results were clear and that they were given a clean bill of health; concerns that they may have had before the procedure were allayed.

…and I would say that the reassurance I got from knowing that, that they have found something and that something that they'd found was not cancerous …I don't know, it was just really reassuring… [P14‐End‐NRC]

##### 3.2.7.2 Monitoring and further investigations

They also talked about ‘being in the system’, knowing that all was fine and that they were being monitored after undergoing endoscopy (I). This included feeling reassured by further investigations for potential causes of developing polyps.

#### 3.2.8 The impact of complex polyp removal on quality of life

Most individuals did not perceive any physical or emotional changes following the procedure and thought that their quality of life remained the same as before.

…the polyp removal did not affect my quality of life… one iota; … the physical side of it… it was fine… it didn't affect my life… whatsoever… [P14‐End‐NRC]

The main changes in quality of life described related to physical functioning and how participants felt within themselves whilst recovering. These changes varied slightly depending on the complexity of the procedure (e.g., how the procedure went) and the time taken to recover. While most commented on short‐term changes to their quality of life, others experienced longer‐term physical and emotional changes that influenced their quality of life.

One participant undergoing endoscopy expressed how their mood improved after removal of the complex polyps (J). This was similar to people who mentioned feeling reassured of the outcomes of their procedure. Individuals for whom there were no major procedure complications mentioned temporary restrictions to their lives in terms of mobility, mood and bowel function, and talked about how they adjusted to these changes. One participant expressed feeling quiet and took time to concentrate on getting better. Another felt low and despite advice to take it easy, pushed herself as she was keen to recover, which intensified her pain.

…the only thing I noticed… was that obviously… I couldn't exercise. I was told not to go out… not to rush… not to go for long walks, I think… I actually went to a birthday party the other side of the country. I drove there. And when I got there, I was in so much pain… that was something that I did… myself… I mean I shouldn't have… I just sort of went too far too fast. I tried to get my life back to normal quicker than I should have… [P28‐End‐NRC]

A greater impact on quality of life was seen in those who had negative outcomes following their procedure. A couple of participants who received a cancer diagnosis talked about changes in their quality of life due to the effects of chemotherapy and their slow recovery. One of these talked about actively looking after his mental health and keeping physically active by resuming his daily exercise routine gently.

…I made sure that I got back into my daily exercise routine gently but definitely doing it every day. I just did that straight away and got that physical fitness quickly. The bowel from the other hand, that was a different matter…it is only just beginning to get back to normal now. Obviously, that's been complicated by 5 weeks after the operation starting a course of chemo which really messes with you as well. Yes, the bowel, the recovery of normal functioning of the bowels is very difficult;… so really several months now since… I am beginning to get back to normal. [P39‐End‐Surg‐NRC]

The other participant undergoing chemotherapy experienced various symptoms, but was not sure whether these were directly associated with the surgery (K). One participant who had to go through emergency surgery following endoscopic removal of his polyp described how difficult it had been to recover both physically and emotionally (L).

#### 3.2.9 Competing factors influencing overall quality of life

Whilst some participants did not notice major changes to their quality of life due to the procedure, they mentioned other competing factors that influenced their quality of life, which included comorbidity, personal circumstances (e.g., family commitments) and COVID‐19 (e.g., bereavement, not being able to socialize, exercise or get timely appointments for health‐related problems) (M).

## 4 DISCUSSION

The current study sought to explore participants' experiences of having a complex polyp removed. These experiences were mainly described through emotional and physical aspects associated with the procedure. They talked about how they viewed their recovery, swift or slow depending on the outcomes of their procedure, and how they felt supported throughout by their families and the healthcare team. Because the majority of participants had an endoscopic procedure and only a limited number had surgery, comparisons between these groups are done with caution. Reflecting back to their experiences, people undergoing endoscopic procedures (apart from exceptional cases) generally talked about having a positive experience, being reassured of positive outcomes and reported none or fewer problems associated with the procedure (pain, additional hospital stay, negative outcomes) that did not seem to negatively impact their quality of life. Those who had surgery (either as the main procedure to remove the complex polyp or due to complications following the complex polyp removal) reported more difficult experiences that were linked to a cancer diagnosis and longer hospital stay, which may have impacted negatively on their health‐related quality of life.

The findings of the present study are consistent with previous research where quality of life seemed to be mainly linked to complications following the procedure rather than the actual procedure.^11^ Positive feelings reported following successful surgical procedures (e.g., elation) have been previously documented.^19^ The present study supports findings from a recent international study investigating patients' colonoscopy experiences that also reported participants worrying about the procedure and its outcome, unpleasant experiences of bowel preparation and finding communications with the healthcare team reassuring around the time of the procedure.^20^

The strengths of this study relate to the recruitment of a varied sample in terms of age, education and location of the procedure. While location of the procedure (NRC or LAC) in our sample is representative of the NMDT service, a slightly greater proportion had an endoscopic procedure within our sample, but this is consistent with there being a majority of endoscopic and fewer surgical procedures that routinely go through the NMDT (P. Jones, personal communication, October 16, 2019). This study adds to the body of knowledge in the field, considering the limited evidence of patients' experiences of complex polyp removal and its impact on quality of life. One of the limitations of the study is recall bias because of the length of time between the complex polyp removal and the interview, and the cross‐sectional nature of the study. This time delay was longer than initially planned because of COVID‐19 pandemic restrictions (pause in procedures as well as delays in amending and setting up the study and receiving post). While there may have been some recall bias, the delay between procedures and interviews meant that participants could reflect on changes in their quality of life. The COVID‐19 pandemic was one of the competing factors that participants reported as influencing their quality of life. It is possible that their perspectives of quality of life have changed as a result of the pandemic and this may have affected their reflections of quality of life after undergoing the complex polyp removal.

Given that most participants went through endoscopy for the removal of their complex polyp, future research could include more people who had surgery as well as more people who experienced complications following either procedure. This would allow confirmation, or otherwise, of the preliminary differences found in quality of life in the present study that appear to be associated with the presence of complications rather than type of procedure. The complex polyp removal service described is currently delivered as part of a structured bowel cancer screening programme in Wales. It would be useful to examine patient experiences across other bowel screening programmes in the United Kingdom.

In the present study, patients' experiences and perspectives of complex polyp removal were generally positive. However, there may be some aspects to consider for improvement such as managing comfort during the procedure and improving communication (e.g., delivering bad news). This study provides insight into what individuals experience and points to potential opportunities for improving patients' experiences of polyp removal by providing support tailored to their needs. Support such as signposting to coping strategies or providing counselling may help people manage worry prior to the procedure and reduce the potential negative impacts of problems experienced following the procedure or a cancer diagnosis on quality of life.

## 5 CONCLUSION

To our knowledge, the present study is the first to provide an understanding of participant's experiences of having a defined pathway for decision‐making and intervention for screen‐detected complex polyp removal and how it affects their quality of life. In general, participants reported a positive experience of the procedure and reassuring interactions with the healthcare team. Any physical and emotional changes to quality of life were usually related to the outcome of the procedure.

## AUTHOR CONTRIBUTIONS

Sunil Dolwani, Kate J. Lifford and Lenira Semedo were involved in developing the study concept and design. Kate J. Lifford and Lenira Semedo, Ardiana Gjini were involved in data acquisition. Kate J. Lifford and Lenira Semedo were involved in data analysis. Kate J. Lifford, Lenira Semedo and Sunil Dolwani were involved in data interpretation. Kate J. Lifford and Lenira Semedo were involved in drafting of the manuscript. All authors contributed to the manuscript substantially by critically revising and approving the final version of the submitted manuscript.

## CONFLICTS OF INTEREST

The authors declare no conflicts of interest.

## Supporting information

Supplementary information.

## Data Availability

The data that support the findings of this study are available from the corresponding author upon reasonable request.
