# Supplementary material for: Participants' experiences of the management of screen‐detected complex polyps within a structured bowel cancer screening programme
Source: Health Expect. 2022 Jul 7;25(5):2355–64. doi: 10.1111/hex.13525 (PMC9615050; doi:10.1111/hex.13525)
Supplement: Supplementary file 1 — Supplementary information. [file HEX-25--s001.docx]

**Interview topics and prompts**

1. **Type of procedure**
   1. What type of procedure did you have?
   2. Where did you have this procedure done?
   3. Were there any alternative options for your procedure?

*Prompts: location, sedation/anaesthetic, open/laparoscopic/endoscopic*.

1. **Experience**
   1. Can you describe your experience of going for your procedure (from being referred after a positive result, to what happened in the following weeks)?

*Prompts: practical issues (travel arrangements, support (e.g. friends/family), bowel preparation, satisfaction with care).*

- 1. Did you experience any problems as a result of your procedure? Please explain. *Prompts: bowel function, diet restrictions, short-term/long-term problems, complications, hospital stay, further treatment.*

1. **Health-related quality of life**

3.1 How would you describe your current quality of life at the moment?

3.2 What influence has having the procedure had on your current quality of life?

*Prompts: mobility, self-care, bowel function (bowel obstruction, incontinence, bloating) usual activities, pain/discomfort, anxiety/depression, procedure outcomes (e.g. cancer diagnosis, complications, further treatment).*

1. **Other**
   1. Is there anything else you think it would be useful to mention about your procedure?
